# Analysis of the Fragile X Trinucleotide Repeat in Basques: Association of Premutation and Intermediate Sizes, Anchoring AGGs and Linked Microsatellites with Unstable Alleles

**DOI:** 10.2174/138920208784340722

**Published:** 2008-05

**Authors:** M.I Arrieta, J.M Ramírez, M Télez, P Flores, B Criado, M Barasoain, I Huerta, A.J González

**Affiliations:** 1 Department of Genetics, Faculty of Science and Technology, University of the Basque Country, Spain; 2 Department of Nursery, School of Nursery, University of the Basque Country, Spain; 3 CESPU, Porto, Portugal; 4 Department of Internal Medicine, Faculty of Medicine, University of Basque Country, Spain

**Keywords:** Fragile X syndrome, FMR1 gene, CGG repeat, FRAXAC1, DXS548, basque country.

## Abstract

Fragile X Syndrome (FXS) is associated with an unstable CGG repeat sequence in the 5’ untranslated region in the first exon of the FMR1 gene which resides at chromosome position Xq27.3 and is coincident with the fragile site FRAXA. The CGG sequence is polymorphic with respect to size and purity of the repeat. Interpopulation variation in the polymorphism of the FMR1 gene and consequently, in the predisposition to FXS due to the prevalence of certain unstable alleles has been observed. Spanish Basque population is distributed among narrow valleys in northeastern Spain with little migration between them until recently. This characteristic may have had an effect on allelic frequency distributions. We had previously reported preliminary data on the existence of FMR1 allele differences between two Basque valleys (Markina and Arratia). In the present work we extended the study to Uribe, Gernika, Durango, Goierri and Larraun, another five isolated valleys enclosing the whole area within the Spanish Basque region. We analyzed the prevalence of FMR1 premutated and intermediate/grey zone alleles. With the aim to complete the previous investigation about the stability of the Fragile X CGG repeat in Basque valleys, we also analyzed the existence of potentially unstable alleles, not only in relation with size and purity of CGG repeat but also in relation with DXS548 and FRAXAC1 haplotypes implicated in repeat instability. The data show that differences in allele frequencies as well as in the distribution of the mutational pathways previously identified are present among Basques. The data also suggest that compared with the analyzed Basque valleys, Gernika had increased frequency of susceptibility to instability alleles, although the prevalence of premutation and intermediate/grey zone alleles in all the analyzed valleys was lower than that reported in Caucasian populations.

## INTRODUCTION

The Fragile X Syndrome (FXS, OMIM 309550) is an inherited form of mental retardation and is linked with a rare fragile site on the long arm of the X chromosome at Xq27.3 (FRAXA). In the vast majority of the affected individuals it is caused primarily by a single type of mutation. This mutation is the unstable expansion of a CGG trinucleotide repeat sequence found in the 5´untranslated region in the first exon of the Fragile X Mental Retardation-1 (FMR1) gene [1].

The CGG sequence is polymorphic with respect to size and purity of the repeat. Based on the size, individuals are classified as having normal alleles (6-54 CGG), premutation alleles (55-200 CGG) and full mutation alleles (>200 CGG). In addition the term intermediate/grey zone alleles has been used to define alleles with sizes at high range of normal alleles (35-54 CGG). Single AGG triplets that are variable in both number and location interrupt the CGG repeat of FMR1 in normal chromosomes. Normally a single AGG interrupts the repeat sequence every 9-10 CGG repeats [2,3].

Massive CGG expansion is the causative mutation in >95% of patients with FXS [3]. This expansion leads to the hypermethylation and consequent inactivation of the gene. Thus, the phenotype is due to the absence of FMR1 encoded protein (FMRP) [3]. Although, only the full mutation is associated with clinical and cytogenetic expression of FXS, premutation carriers have also been associated to distinctive phenotypes [4-6]. In these carriers, the FMR1 gene remains transcriptionally active and FMRP is produced. However it was demonstrated that FMR1 expression is altered for premutation alleles. Specifically, FMR1 mRNA levels were found to be higher than normal despite a reduction in FMRP levels [3]. Surprisingly, functional effects on gene expression may occur even for repeat sizes which have been considered the “normal range”. Recently, we found a correlation between carriers of intermediate/grey zone alleles and premutation associated phenotypes [6-10].

The probability of expansion of the CGG repeats depends upon its own size (reviewed in [11]). Thus, premutation alleles are usually unstable and are subject to further expansion when transmitted by a female, with the potential for expansion proportional to CGG repeat size [12, 13]. All premutation alleles (>100 repeats) expand into the full mutation when transmitted through a female. However, the threshold of instability is no clear and intermediate/grey zone alleles show an uncertain stability upon transmission [14]. In relation to this, transmission of these alleles through males was less stable than that through females [15].

Another molecular characteristic associated with instability of the repeat is the AGG interspersion. These interruptions have been proposed to stabilize the repeat preventing it from expansion. The loss of the most distal 3´ end AGG interspersion is one possible mechanism leading to instability of the repeat [2, 15-17]. It has also been proposed that the 5´end of the CGG repeat, specifically the position of the first AGG interruption, might be another factor for instability [16-18]. Bodega and Zhong *et al*. found that the loss of AGG interruptions also occurred in some intermediated/grey zone alleles [7, 19].

Beside the size and purity of the CGG repeat, background haplotype has been implicated in CGG repeat instability through as yet unidentified *cis*-acting factors, presumably located in the FMR1 locus [14]. DXS548 [20] and FRAXAC1 [21] two dinucleotide (CA) repeat markers 150 and 7 Kb respectively proximal to the CGG repeat have been the most characterized marker loci used in association studies. Haplotype construction of these markers has revealed linkage disequilibrium between the normal and stable alleles but also the unstable full mutation and premutation and intermediate/grey zone CGG alleles [14, 19]. 

Therefore, either large alleles or alleles with long tracts of pure CGG repeats principally found on haplotype backgrounds associated with the full mutation have been proposed to be unstable.

Since the length and structure of allele have an influence on its risk of expansion, the prevalence of certain alleles in one population could affect the incidence of the disease [22]. In fact, some populations have been reported to be predisposed to fragile X syndrome [23, 24], whereas others seem to be less prone to this disorder [25]. Our previous cytogenetic and molecular screening for fragile X syndrome among mental retarded people of Basque and no Basque origin obtained from institutions and special schools [26-29] showed an absence of full mutation among Basque sample. Subsequent investigations on FMR1 gene among normal Basque sample showed a low frequency of large alleles and the maintenance of AGG interruptions on them [30]. However, we recently reported that despite these characteristics, different mutational pathways that might lead to fragile X syndrome could be occurring among Basques [31]. The instability factors observed led us to suggest that these alleles could become into larger CGG alleles and, finally, into fragile X chromosomes. 

Spanish Basque population is distributed among narrow valleys in northeastern Spain with little migration between them until recently. This characteristic may have had an effect on allelic frequency distributions and therefore, in the incidence of Fragile X mutation associated diseases.We had previously reported preliminary data on the existence of FMR1 allele differences between two Basque valleys (Markina and Arratia) [32]. In the present work we extended the study to Uribe, Gernika, Durango, Goierri and Larraun, another five isolated valleys enclosing the whole area within the Spanish Basque region. We analyzed the prevalence of FMR1 premutated and intermediate/grey zone alleles, because recent clinical and molecular studies have changed the view that premutated alleles serve only as a source for full mutation alleles in transmission of FXS and that functional and phenotypic effects are not associated with FMR1 repeat size in the high end of the normal range alleles. With the aim to complete the previous investigation about the stability of the Fragile X CGG repeat in Basque valleys, we also analyzed the existence of potentially unstable alleles, not only in relation with size but also in relation with purity of CGG repeat and DXS548 and FRAXAC1 haplotypes.

## MATERIALS AND METHODS

### Blood Samples

Blood samples were obtained from 298 healthy unrelated male individuals of Basque origin, 58 from Uribe, 60 from Gernika, 72 from Durango, 62 from Goierri and 46 from Larraun. The sample constitutes a solid proportion of the unrelated Basque origin population of each valley. Their Basque origin was confirmed by analyzing the ancestry on the basis of two criteria: the place of birth and the surnames. Basque surnames constitute a good criterion because they are very different not only from those of other Spanish populations but also from valley to valley within the Basque country. Therefore an individual is considered autochthonous of one valley if his grandparents and great-grandparents were born in that valley and if his Basque surnames are characteristics of that valley (In Spain, both the father´s and the mother´s surnames are used in a sequential order, so it is easy to ascertain the grandparent´s and/or the great-grandparent´s surnames).

### DNA Analyses

Genomic DNA was extracted from peripheral blood leukocytes according to standard procedures [33].

FMR1 (CGG)n repeat was amplified by PCR as described by [34]. The product was purified using a High Pure PCR Product Purification Kit (Roche Diagnostics) and sequenced by an ABI310 DNA sequencer (Applied Biosystems). Allele nomenclature indicates the number of repeats. The AGG interspersion pattern is described as the number of uninterrupted CGG repeats with a plus sign (+) indicating the presence of an AGG interruption. DXS548 and FRAXAC1 markers near the FMR1 CGG repeat were analyzed. Both of them were CA dinucleotide polymorphisms located ~150 kb and ~7 kb proximal to the repeat, respectively [20, 21]. DXS548 was amplified by PCR as in [21] using the new forward primer designed by [35] and FRAXAC1 as in [36]. The amplification product was run on a 6% denaturing polyacrylamide gel and visualized by silver staining. Allele nomenclature refers to the number of CA repeats, determined by comparison with known size standards. Haplotype construction has been done from the most proximal to the most distal marker, that is, centromere- DXS548-FRAXAC1-telomere.

### Statistical Methods

Homogeneity tests between population groups were adequately analyzed by Pearson’s chi-squared test (χ^2^ test), likelihood test (G^2^ test), Fisher exact test or paired comparison significance test (z-test) as required. Unbiased genetic diversity was analyzed according to [37].

## RESULTS

### FMR1 CGG Repeat Length

The frequency distribution of the FMR1 CGG alleles is shown in Fig. (**1**). The general distribution of the CGG repeat length ranged between 20 CGG and 59 CGG and was similar in the different population groups (p>0.05). The predominant allele was, in all cases, 30 CGG repeats (46.15%-56.25%). Despite this apparent similarity, striking differences were observed. The second most common allele showed different size in each group, being allele 20 CGG in Larraun (18.75%), 23 CGG in Uribe (12.00%), 29 CGG in Durango (12.50%), 31 CGG in Goierri (15.38%) and 32 CGG (10.00%) in Gernika. Two valleys showed statistical differences in the distribution of FMR1 CGG alleles, corresponding to alleles 42 CGG (p<0.05) and 31 CGG (p<0.05) in Goierri and allele 29 CGG (p=0.05) in Durango. The higher percentage of allele 32 CGG present in Gernika was also noteworthy, although it did not reach the significance level (p>0.05). The heteroczygosity of this locus ranged between (63%) in Larraun and (74%) in Goierri. 

The percentage of alleles in the intermediate/grey zone (35-54 CGG) was, (3.12%) in Durango, (7.69%) in Goierri (8%), in Uribe (12.50%), in Larraun and (20%) in Gernika and there are significant differences among valleys (p<0.05), and these are due principally for the percentage of these alleles in Gernika (20%) and Durango (3.12%). This percentage was (10.07%) in the five valleys. The prevalence of intermediate/grey zone alleles in the five valleys analyzed was approximately 1 per 10 in Basque males. One premutation sized allele (59 CGG) was found in Durango, despite being the lowest percentage of intermediate/grey zone alleles. The prevalence of the premutation in the Basque sample analyzed was 1 per 298 in males.

In the previously analyzed valleys the percentage of intermediate/grey zone alleles was (2.45%) in Markina and (12.07%) in Arratia, with four and seven chromosomes respectively. Taken into account the seven analyzed valleys, the frequency of intermediate/grey zone alleles was (7.32%) and the prevalence of this alleles was also approximately 1 per 10 in Basque males. 

### DXS548 and FRAXAC1 Haplotypes 

The distribution of the DXS548 and FRAXAC1 haplotypes in the five analyzed valleys is displayed in Table **1**. Overall 15 different haplotypes were observed in the Uribe, Gernika, Durango, Goierri and Larraun valleys. The range of variation of different haplotypes is from 7 in Larraun to 12 in Durango. The most common one was 20-19 (59.72%-73.91%) in all cases and showed a similar distribution among valleys (p>0.05). The analyses of haplotypes associated to fragile X mutation in Caucasians showed that DXS548-FRAXAC1 haplotype 25-21 or derived (20-21, 21-21, 26-21, 28-21) was evenly distributed among all valleys (p>0.05). The distribution of haplotype 21-18 or derived (20-18, 25-18, 26-18) was very different among the valleys. Regarding haplotype 21-18, it is noteworthy that it is present in just two of the five Basque valleys analyzed: Gernika and Durango, both showing a significant higher frequency of the mentioned haplotype (p<0.001 and p=0.01, respectively).

### AGG Interspersion Pattern in Potentially Unstable Alleles

Table **2** shows the potentially unstable CGG alleles, identified on the basis of the three principal molecular characteristics associated with instability: CGG repeat size, DXS548/FRAXAC1 haplotypes and AGG interspersion pattern. Overall 67 potentially unstable alleles were observed in the valleys.

The analysis of AGG interspersion pattern shows that the distribution of alleles identified on the basis of the length of pure CGG (≥24 CGG) ranged between (3%) in Uribe, Goierri and Larraun and (4.5%) in Gernika and Durango (p>0.05). The analysis of the position of the long tract of pure CGG repeats in the sequence showed differences between alleles with long tracts in the 3' region (65.67%) and alleles with long tracts in the 5' region (34.32%) (p<0.05), suggesting that although both regions suffer instability, the susceptibility to instability is higher in the 3´end of the CGG. The distribution of both type of alleles in the different Basque groups showed that the percentage of alleles with long tracts in the 3' region ranged between (10.44%) in Uribe, Durango and Larraun and (17.91%) in Gernika. The percentage of alleles with long tracts at the 5´end ranged between (0.74%) in Uribe and (13.43%) in Goierri. The population of Uribe had the lower percentage of both types of alleles and there are significant differences between valleys for both (p<0.05). 

The main unstable structure (34.32% of alleles with long uninterrupted CGG) was n+9 (20≤n≤26, where n represents a number of uninterrupted CGG). The distribution of this structure showed a significant difference among valleys (p<0.05). Thus, it represents near to half of the potentially unstable structures identified within the populations from Uribe and Goierri, but has a lower frequency in Gernika and Larraun. The second most common unstable structures (21% and 22%) are 9+9+n (17≤n≤38) and 11+n (22≤n≤25). These structures are evenly distributed among Basque groups (p>0.05). Another relevant structure is 9+n (22≤n≤25). Their distribution among groups is significantly different (p=0.01), being just present within the population from Uribe, Gernika and Durango, but absent in Goierri and Larraun. The remaining unstable alleles show their presence only in Uribe and Goierri.

The study of the AGG interspersion pattern and the size of the CGG showed that none of intermediate/ grey zone alleles lacked AGG interruptions. Only allele 30 in Uribe, allele 29 in Uribe, Goierri and Larraun, allele 28 in Goiherri and allele 22 in Durango lacked AGG interruptions. Among intermediate/grey zone allele 7 had a single interruption, 12 had a double interruption and 4 had a triple interruption. The only one premutation allele had a double interruption.

A direct relation between CGG repeat size and the 3´repeat length was observed. In this way (87.50%) of intermediate/grey zone, premutation alleles have long tracts in the 3´region (with structures 9+9+n, 9+n, 11+n, and others). The frequency of this association was (8.33%) in Uribe, (12.5%) in Durango, (20.83%) in Goierri and Larraun and (25%) in Gernika, and there are significant differences in that frequency between valleys (p<0.05).

The AGG interspersion pattern and DXS548-FRAXAC1 haplotypes analysis showed that the main unstable structure (n+9) is within haplotype (20-19), the second most unstable structures (9+9+n and 11+n) are within haplotypes (25-21 and 20-19) respectively and finally another relevant structure was identified within haplotype (21-18 or derived).

A direct relation between the 3´repeat length and haplotypes (25-21, 21-18 or derived) associated with FXS in Caucasian was found. (52.27%) of alleles with structures (9+9+n, 9+n, 11+n and others) had that association. The frequency ranged between (6.82%) in Uribe and Goierri and (18.18%) in Gernika (p<0.05). However (0%) of alleles with the structure n+9 showed that association. 

Finally, with the evaluation of the size, the AGG interspersion pattern and DXS548-FRAXAC1 haplotypes also showed a direct relation among CGG repeat size, the 3´repeat length and the DXS548-FRAXAC1 haplotypes (25-21, 21-18 or derived) associated with FXS in Caucasian. In relation to this, (58.33%) of intermediate/grey zone alleles had that association. There are also differences among valleys in this relation (p<0.05) and the intervalley frequencies were (4.17%) in Uribe, (8.33%) in Goierri and Larraun, (12.50%) in Durango and (25%) in Gernika. 

## DISCUSSION

The present study involved the analysis of the FMR1 CGG repeat and two flanking microsatellite loci FRAXAC1 and DXS548 in a sample from five natural valleys in the Basque Country. Basques are an ancient population now living in the western Pyrenees Mountains. The origin of the Basques is unknown. Basques speak a language with very distinct characteristic from those of the surrounding populations.

The Basque language, *Euskara, *is an extreme case of a relic language that has survived through thousands of years of continuous linguistic turnover in neighboring regions [38]. According to [39] “Conservation of a distinct language must has been an important factor in maintaining social and genetic identity”. Previous investigations made by our research group reported differences between Basque and non-Basque populations at different levels: dermatoglyphic phenotypic level [40, 41], cytogenetic level [26, 27, 29] and molecular level [30, 42, 43], corroborating the existence of genetic peculiarities in this population.

Interpopulation variation in the polymorphism of the FMR1 gene and, consequently, in the predisposition to fragile X syndrome due to the prevalence of certain unstable alleles has been observed. Basque Country can be geographically subdivided into two main different areas: one coast and mountainous area in the north and one flat area in the south. The former, where actually most of the people with Basque origin live, is characterized by an irregular orography. Mountains are not too high, but they are spread all along this area. The existing rivers, therefore, form different valleys with a very limited communication between them until recent days. Thus, Basque population can be divided into different isolated groups, what may have had an effect on the prevalence of certain unstable alleles and, therefore, on the stability of the FMR1 locus. With the aim of studying this possible effect, in a previous work we have analyzed the factors implicated in CGG repeat instability in two Basque Valleys (Markina and Arratia) and the results obtained showed allelic diversity between the valleys [32]. To complete the previous investigation in the present work we extended the study to another five different isolated population groups from the Basque Country. The data showed that differences in allele frequencies as well as in the distribution of the mutational pathways previously identified [31, 32] are present among Basques.

The general distribution of the CGG repeat allele sizes was similar among the different Basque groups, suggesting a similar evolution of the CGG repeat in all of them. The most frequent allele showed 30 CGG repeats in all cases. However, significant differences were found in the distribution of secondary alleles. The high frequency of allele 20 in Larraun is notheworthy (18.75%), an allele reported almost exclusively among Caucasians [44]. Also, a high percentage of allele 31 was found in the population from Goierri, a high percentage of allele 29 CGG was found in Durango and, interestingly, a high frequency of allele 32 CGG, usually associated to instability [45], was identified within the population from Gernika. In addition, data on heterozygosity values suggest a greater antiquity for the Goierri settlement [46].

Since the size of the allele is an important indicator of its likelihood of expansion (reviewed in [11]), and differences in the CGG length of the alleles were found, we analyzed the distribution of intermediate/grey zone alleles (35-54 CGG) among Basque groups. The frequency of such alleles ranged between (3.12%)% in Uribe and (20%) in Gernika. The higher frequency of intermediate/grey zone alleles in Gernika is also notheworthy. The frequency of such alleles in the previous analyzed valleys is slightly lower in Markina (2.45%) and these frequencies were comparable to Arratia (12.07%) and Larraun (12.50%). If intermediate/ grey zone alleles showed an uncertain stability upon transmission [14,15] and if only overall length of the repeat was considered, the frequency of potentially unstable alleles was higher principally in Gernika, and also in Arratia and Larraun. However, the estimated prevalence of the intermediate/grey zone alleles 1 per 10 in Basque males (7.32%) was lower than that reported in Caucasian populations [2, 35, 47-50]. The prevalence of premutation alleles in the Basque sample analyzed was 1 per 298 in males. The prevalence of premutation alleles (>54 repeats) in the general population was stimated at 1/813 males [51-53]. As the two Basque valleys previously analyzed [32], intermediate/grey zone alleles devoid of AGG interruptions were not found. Only fourth normal alleles, two in Uribe, two in Goierri, one in Larraun and one in Durango were devoid of AGG interruptions.

Analysis of the AGG interspersion pattern showed that the length of uninterrupted CGG is also correlated to the risk of expansion of an allele (reviewed in [37]). A minimum length of pure repeats is suggested to be needed for an allele to show instability. In the Basque population analyzed, the distribution of alleles with long tracts of pure CGG repeats (≥24 CGG) was similar between valleys. If total length of the repeat does not always identify the pool of potentially unstable alleles, short alleles with long tracts of pure CGG may also acquire a certain degree of instability. This finding could indicate that in relation with this data, there is the same pool of potentially stable alleles in the analyzed valleys. The position of uninterrupted CGG suggests that the susceptibility of instability is higher in de 3’ end of the CGG and principally the Gernika valley. In addition, three intermediate/grey zone alleles (in Larraun) and the only premutated allele (in Durango) had ≥24 pure CGG repeats at the 3´end. The same results were obtained in Larraun as previously in Arratia. If according to [45], the larger alleles have been generated by gradual increments of CGG repeats distal to the most 3´interruptions, this data suggest that the 3´pure CGG repeat is a possible factor of instability in the mentioned valleys.

The number and the position of the AGG interspersion pattern may be another important component of repeat structure that stabilizes the repeat during replication [15, 18, 45, 54]. In relation to the number, a study of [45] suggested a higher stability for (9+9+n) structure than for (9+n). In the present study, (20.83%) of intermediate/grey zone and premutation alleles has (9+n) structures and only in three valleys (4.17%) in Uribe and Durango and (12.50%) in Gernika. In relation to the positions [15, 18, 54] suggested that the structure (9+n) is more prone to expansion than the (11+n) structure. If (9+n) was more unstable than (11+n), the incidence of (9+n) in intermediate/grey zone alleles should be higher. In fact, only (12.50%) of intermediate/grey zone alleles was found to have (11+n) structure, and (20.83%) was found to have (9+n) structures. As in the previous study [32] and according with these results, the length of the 3´pure CGG repeat and position of the 5´-most AGG suggested site specificity with regard to where expansion and susceptibility to instability could occur within the AGG pattern in the valleys. This susceptibility appeared preferently in the Gernika valley.

The tracts of uninterrupted CGG repeats can become longer either through a gradual slippage or loss of an AGG triplet. In fact, the linkage disequilibrium observed between certain markers flanking the CGG repeat and the full mutation suggests the existence of both mutational pathways [35, 45]. The most analyzed markers are the microsatellites DXS548 and FRAXAC1, both of them showing CA repeats. In Caucasian populations, two main DXS548-FRAXAC1 haplotypes, 25-21 and 21-18, have been associated to fragile X mutations. Haplotype 25-21 has been extensively associated to the larger (intermediate/grey zone and premutation) CGG alleles regularly interspersed by AGG interruptions, showing an internal structure 9+9+n. These structures were proposed as resistant to the loss of AGG interruptions, progressing slowly to mutations by addition of repeats at the 3’ end [45]. This association was also found in the Basque population, representing 21% of the potentially unstable alleles and it is evenly distributed among the 5 isolated Basque groups. On the contrary, haplotype 21-18 has been extensively associated to asymmetrical structures such as 9+12+9 or 9+10+9 within normal alleles. [45] suggested that these structures were prone to the loss of the 3’ AGG leading to structures 9+n that expanded quickly to the full mutation. No intermediate and even premutation alleles are therefore found within this haplotype or, at least, not in a detectable frequency, what supports the need to analyze other instability factors than total repeat length. It is noteworthy that this haplotype is present just in two of the five valleys: Gernika and Durango, being more represented in Gernika, what is in accordance with the high frequency of allele 32 CGG identified in this population group. 

Interestingly, the most prevalent unstable structure among Basques is n+9, a structure lacking the 5’ most AGG. [55] suggested that structures lacking the 5’ most AGG might progress rapidly to the full mutation after the loss of the 3’ AGG leading to pure CGG repeats. Among Basques, structures such as n+9 or pure CGG were identified within haplotype 20-19. Analysis of the distribution of this mutational pathway among population groups showed that it represents near a half of the potentially unstable structures identified in the populations from Uribe and Goierri, representing a continuous geographical route of this pathway. It is absent however in Durango and Larraun, where the potentially unstable alleles show long uninterrupted CGG repeats at the 3' end.

The data from this work suggest that despite the relatively small geographical area where Basque tribes settled, and their common ethnic, linguistic and cultural origin, at the FMR1 locus the Basque population seems to be genetically heterogeneous. The data also suggest that compared with the analyzed Basque valleys, Gernika had increased frequency of susceptibility to instability alleles, although the prevalence of premutation and intermediate/grey zone alleles in all the analyzed valleys was lower than that reported in Caucasian populations. Haplotype analysis showed that each one of the mutational pathways identified resulted most probably from a unique or few mutational events [31]. Therefore, the study of their distribution greatly facilitates the analysis of population group relationships. The present report shows that these mutational pathways are geographically patterned among Basques, probably indicating a route of expansion for them. In summary, the heterogeneity observed in Basques can be attributed to the result of gene flow and genetic drift within the isolated Basque groups.

## Figures and Tables

**Fig. (1):**
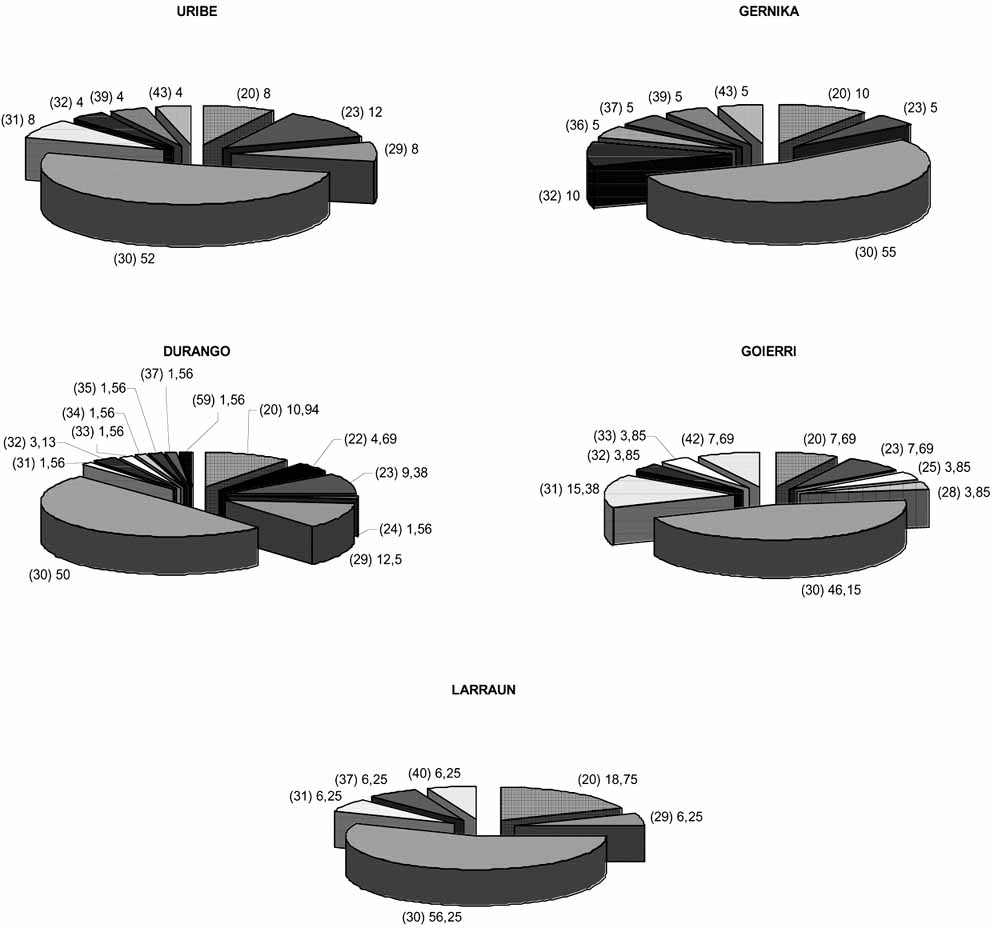
Distribution of FMR1 CGG alleles in Basque Valleys. The first number (in bracket) is the allele size and the second its percentage frequency.

**Table 1.** Distribution of the DXS548-FRAXAC1 Haplotype Frequencies

Haplotype	Uribe		Gernika		Durango		Goierri		Larraun		Total	
	N	%	N	%	N	%	N	%	N	%	N	%
28-21^a^	1	1,72	1	1,67	1	1,39	1	1,61	1	2,17	5	1,68
26-21^a^	2	3,45	1	1,67	2	2,78	1	1,61	1	2,17	7	2,35
26-18^b^	1	1,72	1	1,67	1	1,39					2	0,67
25-21	2	3,45	3	5	4	5,56	3	4,83	2	4,35	14	4,70
25-19	1	1,72					2	3,23			3	1,01
25-18^b^	1	1,72	3	5	1	1,39	1	1,61			5	1,68
24-21			1	1,67	1	1,39					3	1,01
21-21^a^	1	1,72			1	1,39	1	1,61			4	1,34
21-19	2	3,45	3	5	12	16,67	9	14,52	2	4,35	28	9,40
21-18			8	13,33*	3	4,17*					11	3,69
20-21^a^	2	3,45					1	1,61	2	4,35	5	1,68
20-20					1	1,39			2	4,35	3	1,01
20-19	42	72,41	37	61,67	43	59,72	41	66,13	34	73,91	197	66,11
20-18	3	5,17			2	2,78	2	3,23	2	4,35	9	3,02
20-17			2	3,33							2	0,67
Total	58	100	60	100	72	100	62	100	46	100	298	100

a Haplotypes supposed to be derived from 25-21 through slippage and/or recombination.

b Haplotypes supposed to be derived from 21-18 through slippage and/or recombination.

* Statistically significant differences among population groups.

**Table 2.** Potencially Unstable CGG Alleles in the Different Basque Groups

CGG	Haplotype	Sequence	Uribe	Gernika	Durango	Goierri	Larraun	N
**25-21 pathway**		**(9+9+n)**						
59	21-21	9+9+38			1			1
42	25-21	9+9+22				2		2
37	20-21	9+9+17					2	2
37	25-21	9+9+17		3	1			4
30	25-21	9+9+19	2			1	2	5
**21-18 pathway**		**(9+n)**						
43	25-18	9+10+12+9	1	3				4
35	25-18	9+25			1			1
34	26-18	9+24			1			1
32	21-18	9+22		2	1			3
**20-19 pathway**		**(n+9)**						
36	20-19	26+9		3				3
31	20-19	21+9	3		1	6	2	12
30	20-19	20+9		1		1		2
30	20-19	30	1					1
29	20-19	29	1			1	1	3
28	20-19	28				1		1
22	20-19	22			1			1
		**(11+n)**						
40	20-19	11+28					3	3
33	20-19	11+21			1	2		3
32	20-19	11+20	2	4	1	2		9
**Other**								
43	20-19	10+9+22	1					1
42	20-19	10+11+19				3		3
31	20-19	10+20	1			1		2
